# Peri-parturient hypocalcemia in goats: Clinical, hematobiochemical profiles and ultrasonographic measurements of postpartum uterine involution

**DOI:** 10.14202/vetworld.2021.558-568

**Published:** 2021-03-02

**Authors:** Yasmin H. Bayoumi, Amany Behairy, Asmaa A. Abdallah, Noura E. Attia

**Affiliations:** 1 Department of Animal Medicine, Faculty of Veterinary Medicine, Zagazig University, Egypt; 2 Department of Physiology, Faculty of Veterinary Medicine, Zagazig University, Egypt; 3 Department of Theriogenology, Faculty of Veterinary Medicine, Zagazig University, Egypt

**Keywords:** does, hypocalcemia, postpartum, ultrasonography, uterine involution

## Abstract

**Background and Aim:**

Hypocalcemia in goats occurs around the time of parturition and is caused by decreasing level of calcium less than 10 mg/dL. This investigation characterized the hematological and biochemical profiles of peri-parturient hypocalcemia in goats and study the effects of hypocalcemia on uterine involution during the postpartum period on day 0 and then weekly postpartum (day +7, +14, +21, +28, +35, till +42).

**Materials and Methods:**

Forty-five polyparous native breed does age 3-5 years and weighing 40–60 kg were assigned to control and hypocalcemia groups based on their health history, clinical and biochemical findings. The control group included 10 clinically healthy pregnant does, and the hypocalcemia group included 35 late pregnant does that suffered from anorexia, weakness, muscle tremors of the hind limbs, and an inability to stand. Clinical examination and blood sampling in both groups were performed approximately 14 days before the expected time of kidding (day −14), at kidding day (day 0), on day +21, and +42 postpartum. Sonographic measurements were used to monitor uterine involution on day 0 and then weekly once the animal was postpartum (day +7, +14, +21, +28, +35, till +42) in both groups.

**Results:**

Results of sonographic measurement revealed that the hypocalcemia group had a greater (p<0.05) size of the uterus as assessed transrectally and retarded uterine involution when compared with the control group. Laboratory findings revealed that does in both groups showed lower red blood cells, packed cell volume%, and hemoglobin (Hb) concentrations on day −14 before kidding and day 0 when compared with postpartum measurements. A significant increase (p<0.05) in total leukocyte counts, neutrophil, serum glucose, and cortisol levels with a significant decrease (p<0.05) in lymphocytes. Significant decreases (p<,0.05) in serum calcium, phosphorous, vitamin D, and total antioxidant capacity levels with significant (p<,0.05) increases in serum malondialdehyde were recorded on day 0 and day −14 in the hypocalcemia group when compared with the control group.

**Conclusion:**

This investigation provides evidence that hypocalcemia in does causes both metabolic and oxidative stress during peri-parturient periods while also retarding uterine involution during postpartum periods.

## Introduction

The practice of goat rearing has gained recent economic interest worldwide as goats are easily able to adapt to a wide variety of environments. Goats are unique as they are able to adapt their metabolic and blood parameters according to the physiological life phase [1]. Successful management of the animal during the peri-parturient period is vital for the health of the doe and to ensure a positive economic impact.

The peri-parturient period is defined as the 6-8-week period centered around parturition. During this critical phase, several metabolic changes and adaptations to the new physiological status of the animal occur [2], making the female susceptible to many metabolic diseases due to the failure to meet nutritional requirements during the late stage of pregnancy and early lactation [3]. The most common metabolic disorders in ewes and does are peri-parturient hypocalcemia, pregnancy toxemia, and hypomagnesemia [4].

Hypocalcemia is an acute or subacute metabolic disease that typically occurs in ewes and does several weeks before and after parturition. It occurs when blood calcium levels fall to 10 mg/dL or less in different species, including cattle and goat [4], and the animal is unable to reestablish concentrations of Ca in blood through intestinal absorption or bone resorption [5]. Hypocalcemia occurs due to low dietary calcium and vitamin D supplementation and an improper Ca and P ratio (2:1) [6]. The risk factors associated with hypocalcemia include species, breed, age, sex, and stage of production [6,7], and it affects cattle primarily and to a lesser extent small ruminants [8], aged does (4-6 years), those pregnant with multiple feti, and dairy goats capable of heavy lactation [9].

Hypocalcemia in goats typically results in hyperesthesia and tetany, more so than the classic flaccid paralysis observed in dairy cattle. It is characterized clinically by loss of appetite, lethargy, an inability to move, recumbency, subnormal rectal temperature, diminished pupillary light reflex, and a relaxed anal sphincter [10]. Hypocalcemia can be the driving factor behind other diseases such as retained placenta and metritis. In addition, it increases the risk of dystocia, particularly during the transition period [11].

The puerperium period is defined as uterine involution and the function of ovaries, making the animal capable of receiving a new pregnancy. In small ruminants, uterine involution occurs when the diameter of the uterus decreases to the same normal size as observed in the normal estrus cycle [12]. The time of uterine involution in goats is recorded between the 3^rd^ and the 10^th^ days after kidding. It is illustrated by decreased uterine weight and length and a reduction in the uterus size after birth [13]. Puerperal disease plays an essential role in the retardation of uterine involution in dairy goats. Several studies have shown that puerperal diseases increased in dairy cows that suffered from metabolic diseases during the puerperium period [14].

However, to the best of our knowledge, no investigations have demonstrated the relationship between hypocalcemia and uterine involution in goats. Thus, the purpose of this investigation was to assess the effect of peri-parturient hypocalcemia on the hematobiochemical profile, the occurrence of dystocia, uterine involution, reproductive performance, and the prevalence of metritis during the postpartum period in goats.

## Materials and Methods

### Ethical approval

The study was approved by the ethical committee of Zagazig University in accordance with the guidelines of the National Institutes of Health for the Care and Use of Animals.

### Study period and location

The study was conducted from July 2018 to March 2019 at Veterinary Teaching Hospital, Faculty of Veterinary Medicine, Zagazig University, Egypt.

### Animals grouping

The study was conducted on 45 polyparous native breed does, aged 3-5 years and weighing 40-60 kg. Ten clinically healthy does from a private farm located in the Sharkia Province, Egypt, were used after synchronization of their estrus and pregnancy (control group). Thirty-five late pregnant does were admitted to the Veterinary Teaching Hospital, Faculty of Veterinary Medicine, Zagazig University, Sharkia Province, Egypt, for investigation and/or to ensure their pregnancy and soundness of the feti, with a previous history and continuous complaint of anorexia, weakness tremors in muscles of the hind limb, inability to stand, frequent standing on the knee joints, and recumbency (hypocalcemia group). All cases included in this study did not receive any calcium therapy except two of seven cases suffering from dystocia. We exert great effort in observation of the cases and follow-up without administration of calcium sources to hypocalcemic cases but when the case administer calcium, it excluded from the study. Determination of ketone bodies in urine samples was routinely estimated using Comburg urine strips (Boehringer Mannheim, Germany) test.

### Synchronization of estrus and pregnancy in the control group

Ten clinically healthy does from the private farm where used, does were shown to have normal estrus activity before the beginning of the study. Synchronization of the estrus was obtained by placing sponges containing 40 mg fluorogestone acetate (FGA; Ceva, France) as a source of intravaginal progesterone. Sponges were left intravaginally for 5 days. The animals received an injection of prostaglandin F2a (PGF2a; 12.5 mg, i.m.; Pharmacia and Upjohn S.A.) on the same day of sponge removal [15]. Goats were mated naturally after the PGF2a injection using fertile bucks. Breeding dates were recorded, and pregnancy status was detected by ultrasonography 45 days post-mating.

### Management and feeding regime

The control group was fed a balanced ration containing 16% protein and 70% TDN. Each animal was provided a daily commercial concentrate mixture (2% of body weight) consisting of 30% crushed maize, 35% wheat bran, 32% and cottonseed cake, supplemented with vitamins and minerals. Rice straw, green fodder (Egyptian clover, *Trifolium alaxandrinum*), darawa, and maize fodder were offered when available. The complete ration was offered through two equal portions twice daily. The hypocalcemia group was reared by stockmen in villages in the vicinity of Zagazig city, Sharkia Province, and after taking history, the feeding regime for this group was nearly similar, all cases were reared under harsh environment. Rational natural pasture (green herbage, grass, and remnants of plant, berseem, and darawa were utilized when available) with or without concentrates. Rice straw was available *ad libitum* during the night.

### Clinical examination

All animals included in the investigation were subjected to a thorough clinical examination, including the monitoring of vital signs, rectal temperature, respiration, and heart rate, and ruminal contractions. Each of the above was measured as described by Dirksen *et al*. [16]. Age, gestational age, parity, presenting signs, and previous illness were also recorded.

### Ultrasonographic examination

Pregnant does were examined through an ultrasound machine (Esaote Mylab, Netherland) using a convex transducer of frequency 2.5-5.5 MHz for determining the age, the number of feti, and viability by monitoring the fetal heartbeats. The transducer was applied to the lower abdomen of the goat while the animal was in a lying position (on the sides of mammary glands).

Uterine involution was monitored using a trans-abdominal probe through a 2.5-5.5 MHz frequency on day 0 (day of delivery) and day 7 postpartum, then weekly postpartum (14, 21, 28, 35, till 42) days by transrectal probe operating at 8 MHz. The doe was secured in a standing position. The transducer was introduced into the rectum and moved medially and laterally to record the length and width of the postpartum uterus, as reported by Medan and El-Daek [17]. Uterine involution was monitored by measuring of uterine thickness diameter at the whole and by recording the uterine contents as caruncles and the rested fluids till disappeared on day 7 and day 12 postpartum in the control and hypocalcemia groups, respectively.

### Blood sampling

Approximately 14 days from the expected date of parturition (day −14), at parturition (day 0), and on day +21 and +42 postpartum (most of the owners refused weekly collection of blood), three blood samples were collected from each animal through jugular vein puncture. The 1^st^ blood sample (7 mL) was collected in plain tubes without anticoagulant and remained in sloped position for 30 min to clot at room temperature before it was centrifuged at 3000 rpm for 15 min and finally serum separated and stored at −20°C for later analysis of biochemical parameters. The second blood sample (2 mL) was collected into dipotassium ethylenediaminetetraacetic acid (K2-EDTA) coated tubes for hematological analysis. The third blood sample (1 mL) was collected in sodium fluoride-containing tubes for glucose estimation [18].

### Hematobiochemical screening

Complete blood picture was determined using an automated blood cell analyzer (Sysmex XT-2000iV, Kobe, Japan). Blood glucose, total serum proteins, albumin (ALb), aspartate aminotransferase (AST), alanine aminotransferase (ALT), alkaline phosphatase (ALP), blood urea nitrogen (BUN), creatinine (Cr), calcium (Ca), phosphorus (P), and vitamin D were measured using commercial diagnostic kits obtained by Biomerieux, and Spinreact, Spain. Serum globulins were calculated mathematically by subtracting albumin from total proteins. Serum cortisol was measured using cortisol enzyme-linked immunosorbent assay kit [Biovision, USA (Cat. No. K7430-100)]. Malondialdehyde (MDA) was evaluated by ELISA kit (Cusabio Biotech Co. Ltd., China) using the methods of Ohkawa *et al*. [19]. Serum total antioxidant capacity (TAC) was determined using TAC assay kit [OxiSelect™, San Diego, CA, USA (Cat. No. STA-360)] according to Cerutti and Trump [20].

### Statistical analysis

All data were analyzed using an analysis of variance using a statistical software program (SPSS for Windows, version 16.0, SPSS Inc., Chicago, IL). Duncan’s *post hoc* test was applied to determine the level of significance between groups. Results are expressed as mean±standard deviation (SD), and significance was accepted at p<0.05.

## Results

### Incidence and clinical findings

The incidence of hypocalcemia was the highest in does over 3 years of age, occurring at a rate of 60% (21 of 35). The occurrence was 65.7% (23 of 35) in does with more than 2 feti and 62.8% (22 of 35) in does with a higher parity. During the follow-up period during the pregnancy in the two groups, during birth, dystocia cases were recorded in the hypocalcemia group with a rate of 20% (7of 35). The retained placenta and metritis within a percent of 8.5% (3 out of 35) as explained in Table-1.

**Table-1:** The difference between two groups regarding dystocia, litter size (twins rate or triple rate), retained placenta%, and still birth of newborn kids%.

Parameter	Control group	Hypocalcemia group
Dystocia %	0%	(7/35) 20%
Retained placenta %	0%	(3/35) 8.5%
No. of kids born	22 from 10 parturient goats	97 from 35 parturient goats
Litter size (twins %-triples %)	Twins % (72.7%, 16 from 8 parturient goat) Triples % (27.2%, 6 from 2 parturient goats)	Twins % (16.5% 16 kids from 8 parturient goat) Triples % (83.5% 81 kids from 27 parturient goats)
Still birth of newborn kids %	3/22 (13.6%)	30/97 (30.9%)

There were seven parturient goats of the hypocalcemia group suffered from dystocia, two of them caused by primary uterine inertia and dealing with them by administrating calcium borogluconate (50 mL, slowly intravenous) with estradiol benzoate 5 mg (folone, 3-5 ampoules, intramuscular). The cause of dystocia in the five rested goats was incomplete cervical dilatation (ring womb), dealing with them by administration of prostaglandin F2a (2 mL Estrumate, MSD, Germany, intramuscular) with estradiol benzoate as mentioned above. Placenta is considered retained 24 h after birth, giving the goats with retained placenta oxytocin hormone (Pitocin, Bioveta, Netherlands, 3-5 mL for 2 days, intramuscular) and applying oxytetracycline tablets 2-4 g intrauterine (2 tablets/day/4 days).

Goat experiences hypocalcemia during late pregnancy associated with rapid calcium loss to the developing fetus for bone mineralization. Other species can experience hypocalcemia at or near the time of peak lactation. Based on the limited information available regarding goats, it seems that dairy breed goats are potentially prone to all three manifestations of hypocalcemia. However, goats under our study were not reared for milk production at all, and the maximum ambition is to give offspring. Moreover, in such circumstances, the weaning usually occurs between 2 and 3 months of age without extra milk for collection.

Vital signs in both groups are shown in Table-2. A significant (p<,0.05) increase in body temperature in the control group was recorded on day −14 reaching the peak on day 0, then begin to decline on day +21 and +42 (all changes were within normal physiological limit) while in the hypocalcemia group, a significant (p<,0.05) decrease in body temperature was recorded on day -14 and day 0 followed by significant increase (p<,0.05) on day 21 and day 42. A significant increase in heart rate (p<,0.05) was recorded on day 0 and day −14 compared with day +21 and +42 in both groups, while a significant increase (p<,0.05) in heart rate was recorded on day 0 and day −14 in the hypocalcemic group when compared with the control group. A significant decrease (p<,0.05) in ruminal movement was recorded in the hypocalcemia group when compared with the control group.

**Table-2:** Vital signs monitoring in the control group (Group 1) and hypocalcemia group (Group 2).

Clinical signs	Groups	−14 day	0 day	21 day	42 day
Body temperature °C	1	39.81±0.17^bA^	40.03±0.18^aA^	39.63±0.09^cA^	39.47±0.22^d^
	2	38.75±0.79^bB^	38.45±0.71^bB^	39.25±0.58^aB^	39.27±0.49^a^
Respiration rate/min.	1	24.10±2.23^b^	26.40±2.07^a^	22.30±1.89^c^	22.20±1.40^c^
	2	24.63±1.97^b^	26.86±1.82^a^	22.29±1.41^c^	22.40±1.31^c^
Heart rate/min.	1	82.40±5.36^bB^	87.20±2.86^aB^	74.50±4.28^cB^	74.80±3.74^c^
	2	99.54±11.20^aA^	101.97±8.42^aA^	84.29±12.17^bA^	77.03±9.20^c^
Ruminal movement/2 min.	1	2.40±0.52^A^	2.00±0.00^A^	2.40±0.52^A^	2.30±0.48^A^
	2	0.20±0.41^cB^	0.14±0.36^cB^	0.80±0.87^bB^	1.34±0.80^aB^

Values with different superscripts ^a, b, c,^ ^and^ ^d^ within the same raw differ significantly (p<0.05) among periods; values with superscripts ^A^ ^and^ ^B^ differ significantly (p<0.05) between the two groups

Regarding the clinical findings in the hypocalcemia group, the severity of clinical signs during the study periods was correlated to the drop in the level of serum calcium. The initial symptoms included hyperesthesia, fine twitching of the ears, lips, and eyelids, muscle tremors (especially in the hindlimbs), progressing anorexia, weakness, ruminal stasis and constipation with hard feces, mild ruminal tympani, and coldness of the ear, mouth, and the skin was also observed. Following these observations, the does were unable to stand. Some were able to stand on knee joints or sternally recumbent with the extension of one hind leg, sometimes with sternal recumbency and the head and neck directed toward the flank. In addition, two cases died during the experiment period, but they did not include in the study.

### Ultrasonographic findings

**Figure-1:**
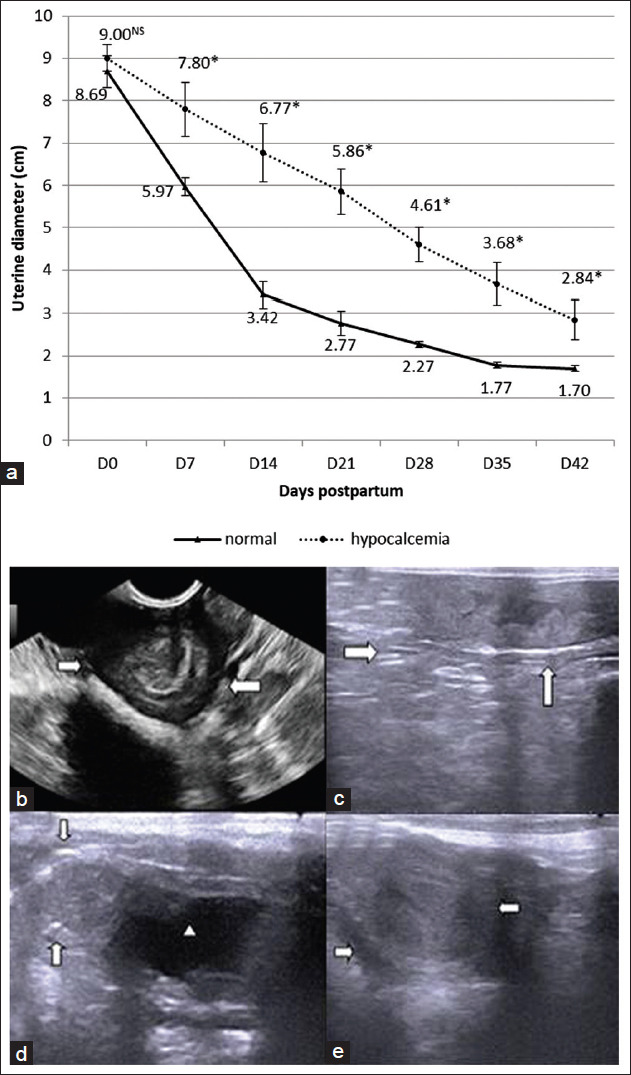
(a) Mean ultrasonographic uterine diameter in the control and hypocalcemia groups on days 0, 7, 14, 21, 28, 35, and 42 postpartum, values with superscripts (*) differ significantly (p<0.05) between the two groups; (b) ultrasonographic image of the postpartum uterus by the transabdominal probe in normal doe at kidding day, the external borders of the uterus are lineated by a white arrow while (c) ultrasonographic image of the postpartum uterus in the control group on day 7; (d) ultrasonographic images of the postpartum uterine horn by the transrectal probe on day 35 after kidding in normal does, The external borders of the uterus are lineated by the white arrow, arrowhead referred to anechoic urinary bladder as a guide, while (e) in hypocalcemic doe on day 35 after kidding, white arrows refer to the external outlines of the uterus.

On day −14, before kidding, ultrasonographic findings in both groups regarding ages, numbers of feti, and the viability of feti by monitoring the heartbeats were applied. All pregnant goats in the present study were pregnant with 2 or 3 feti. The mean of postpartum uterine horn diameters in both groups measured by ultrasound is illustrated in Figure-1a.

On day 0, the mean diameters of the postpartum uterine horn were 8.688±0.369 and 9.002±0.313 in the control and hypocalcemic groups. A rapid decrease in uterine horn diameter was recorded until they reached 5.968±0.216 cm in the control group. In contrast, in the hypocalcemia group, a slight decrease in mean diameters of uterine horns was recorded to 7.802±0.636 cm on day 7 postpartum. Mean diameters of postpartum uterine horns continue in reduction rapidly in the normal group until they reached 1.696±0.074 on day 42, but in the hypocalcemia group, the reduction progressed slowly until they reached 2.840±0.470 on day 42 in comparison to the control group.

Ultrasonographic imaging of the uterine horn on delivery day in both groups showed the presence of anechoic rested fetal fluids, hyperechoic tissue debris, and persistent enlarged hyperechoic caruncles (Figure-1b). The changes in the outer circumference of uterine horns in the control group revealed a higher rate of reduction and disappeared caruncles on day 7 postpartum when compared with the hypocalcemia group, as shown in Figure-1c. During the follow-up period using a B mode ultrasound machine within puerperium period in the control group, a rapid decrease in diameter and, what appeared to be, a completely closed uterus with an empty lumen were observed on day 35 after delivery (Figure-1d). However, uterine horn diameters in the hypocalcemia group showed a slightly decreased size when compared with normal does on day 35 after delivery (Figure-1e).

In the control group, postpartum uterine diameter decreased more than 50% in the first 14 days within a high significance than the hypocalcemic group. Our results revealed that uterine involution in normal occurs during a short period and regresses earlier when comparison with the hypocalcemia group.

### Hematobiochemical findings

Results of hematological parameters are presented in Table-3. Both the control and hypocalcemia groups showed lower red blood cells (RBCs), packed cell volume (PCV%), and Hb concentration on day 14 before kidding and day 0 when compared to day +21 and +42. While a significant decrease (p<,0.05) in PCV% was recorded in the hypocalcemia group compared to controls. Non-significant changes were recorded in mean corpuscular volume, mean corpuscular hemoglobin (MCH), and MCH concentration either between groups or during different periods.

**Table-3:** Hematological profile in control group (group 1) and hypocalcemia group (group 2).

Parameters	groups	-14 day	0 day	21 day	42 day
RBCs (× 10^6^/μL)	1	7.223±0.02^b^	7.567±0.45^b^	8.553±0.12^a^	8.867±0.37^a^
	2	7.100±0.20^b^	7.453±0.03^b^	8.377±0.11^a^	8.600±0.46^a^
PCV(%)	1	24.16±0.12^d^	25.22±0.23^cA^	27.80±0.20^b^	28.65±0.67^a^
	2	23.80±0.34^b^	24.53±0.15^bB^	27.13±0.65^a^	28.00±1.00^a^
Hb (gm%)	1	8.120±0.02^b^	8.410±0.05^b^	9.400±0.46^a^	9.670±0.32^a^
	2	8.033±0.31^b^	8.420±0.03^b^	9.100±0.66^a^	9.400±0.20^a^
MCV (fl)	1	33.45±2.11	33.33±2.11	32.50±0.02	32.31±0.85
	2	33.52±1.02	32.91±0.93	32.39±0.77	32.56±0.83
MCH (pg)	1	11.24±0.50	11.11±0.53	10.99±0.53	10.91±0.53
	2	11.31±0.40	11.30±0.43	10.86±0.71	10.93±0.54
MCHC (%)	1	33.61±0.98	33.35±1.04	33.81±1.18	33.75±1.16
	2	33.75±1.12	34.33±1.03	33.54±0.88	33.57±0.83
WBCs (× 10^3^/μL)	1	10.17±0.30^b^	11.20±0.03^a^	9.220±0.04^c^	8.200±0.36^d^
	2	10.27±0.01^ab^	11.33±0.72^a^	9.500±0.87^bc^	8.640±0.04^c^
Neutrophil(%)	1	44.29±1.34^ab^	45.63±0.87^aB^	41.19±2.67^bc^	39.59±1.98^c^
	2	45.89±2.46^ab^	48.00±1.25^aA^	42.43±2.21^b^	42.09±2.57^b^
Lymphocyte(%)	1	51.26±1.61^b^	50.96±1.09^bA^	54.58±0.97^a^	56.12±2.49^a^
	2	49.97±1.22^bc^	49.06±0.79^cB^	53.17±2.47^ab^	53.47±1.04^a^
Eosinophil(%)	1	2.78±0.17^a^	2.07±0.17^bA^	2.93±0.19^a^	2.79±0.35^a^
	2	2.73±0.23^a^	1.57±0.15^bB^	2.98±0.18^a^	2.96±0.26^a^
Monocyte(%)	1	1.66±0.11^a^	1.35±0.10^b^	1.31±0.11^b^	1.49±0.18^ab^
	2	1.42±0.13	1.36±0.14	1.42±0.09	1.49±0.15
Basophil(%)	1	0.000±0.00	0.000±0.00	0.000±0.00	0.000±0.00
	2	0.000±0.00	0.000±0.00	0.000±0.00	0.000±0.00

Values with different superscripts (a, b, c&d) within the same raw differ significantly (p<0.05) among periods; values with superscripts (A&B) differ significantly (p<0.05) between the two groups

A significant (p<,0.05) increase in total leukocyte counts (TLCs) was recorded on day −14 and reached a peak on day 0, before declining by day +21 and +42 in both the control and hypocalcemia groups. There were no significant differences in TLCs between groups. A significant (p<,0.05) increase in total neutrophils with a significant decrease (p<,0.05) in lymphocytes was recorded on day 0 and day −14 compared with day +21 and +42 in both groups. The highest increase in neutrophil and the lowest values in lymphocytes were reported on day 0. Such alterations were significant (p<,0.05) in the hypocalcemia group when compared to control. A significant decrease (p<,0.05) in eosinophil and monocyte was recorded on day 0 compared to the remaining periods in both the control and hypocalcemia groups. A significant increase (p<,0.05) in eosinophils was recorded on day 0 in the control group when compared with the hypocalcemic group, and a significant increase (p<,0.05) in monocytes was recorded on day −14 when compared to the control group.

Biochemical parameters are presented in Table-4. A significant increase (p<,0.05) in serum glucose levels was recorded on day 0 and day −14 compared with day +21 and +42 in both groups, with the highest level of glucose on day 0. The increases in glucose were more evident in the hypocalcemia group. There was a significant decrease (p<,0.05) in serum total protein and globulin levels on day −14 and day 0 when compared with day +21 and +42 in both groups, with the lowest values observed in the hypocalcemia group. There was a non-significant change in serum AST and ALT levels on day −14, day 0, and day +21 in both groups. Serum ALP level recorded a significant (p<,0.05) increase on day −14 followed by day 0 when compared with day +21 and +42 in the control and hypocalcemic groups. A significant (p<,0.05) increase in serum ALP was recorded on day −14, day 0, and day +42 in the control group.

**Table-4:** Biochemical profile in control group (group 1) and hypocalcemia group (group 2).

Parameters	Groups	-14 day	0 day	21 day	42 day
Glucose	1	60.00±7.21^b^	75.00±5.57^a^	50.00±5.00^bc^	48.00±5.57^c^
	2	65.00±5.57^b^	78.00±7.55^a^	62.00±7.21^b^	54.00±5.29^b^
Total protein (g/dL)	1	5.180±0.18^b^	5.210±0.11^b^	6.470±0.53^a^	7.000±1.11^a^
	2	5.000±0.23^b^	5.130±0.25^b^	6.230±0.48^a^	6.540±0.52^a^
Albumin (g/dL)	1	3.370±0.41	3.070±0.23	3.560±0.78	3.670±0.32
	2	3.400±0.62	3.180±0.49	3.530±0.27	3.540±0.88
Globulin (g/dL)	1	1.810±0.15^b^	2.140±0.16^b^	2.910±0.18^a^	3.330±0.46^a^
	2	1.600±0.56^c^	1.950±0.06^bc^	2.700±0.30^ab^	3.000±0.70^a^
AST (U/L)	1	88.00±11.14	93.00±11.79	90.00±10.00	85.00±10.00
	2	90.00±13.23	95.00±15.00	90.00±13.23	85.00±11.14
ALT (U/L)	1	25.88±0.06	29.13±2.14	25.00±5.00	24.00±3.61
	2	29.33±5.05	32.00±4.58	28.87±1.17	26.00±4.58
ALP (U/L)	1	74.12±1.50^aA^	65.28±1.10^bA^	60.53±1.30^c^	61.68±2.40^cA^
	2	67.07±2.3^aB^	63.30±0.60^bB^	57.66±1.52^c^	51.60±3.36^dB^
Urea (mg/dL)	1	28.15±0.86^cA^	40.97±1.43^a^	33.61±0.53^b^	31.10±2.26^b^
	2	26.00±1.00^cB^	38.67±0.76^a^	31.00±2.65^b^	29.93±1.40^b^
Creatinine (mg/dL)	1	1.120±0.03^a^	1.030±0.04^b^	0.810±0.05^c^	0.610±0.06^dB^
	2	1.200±0.30^a^	1.080±0.03^ab^	0.900±0.10^ab^	0.770±0.03^bA^
Calcium (mg/dL)	1	8.110±1.12^bA^	7.000±0.50^cA^	7.117±0.46^c^	9.020±0.33^a^
	2	6.333±0.50^bB^	4.900±0.41^cB^	6.100±0.70^b^	8.000±0.62^a^
Phosphorus (mg/dL)	1	4.130±0.07^bA^	3.520±0.09^cA^	3.550±0.29^c^	4.780±0.11^a^
	2	3.860±0.09^bB^	3.250±0.07^cB^	3.300±0.50^c^	4.350±0.53^a^
Vit D (g/dL)	1	22.00±1.50^abA^	25.66±2.76^aA^	20.18±0.54^b^	19.00±1.70^b^
	2	18.00±1.00^B^	18.04±0.83^B^	20.23±0.58	20.61±1.66
Cortisol (ng/mL)	1	10.00±2.00^b^	14.00±2.65^a^	8.000±1.00^b^	7.600±0.50^b^
	2	11.00±3.61^b^	16.00±3.61^a^	9.000±1.00^b^	8.600±0.79^b^
MDA (nmol/mL)	1	16.23±1.05^bB^	18.12±1.78^aB^	14.79±1.95^cB^	13.42±0.31^dB^
	2	20.12±1.12^cA^	23.16±0.84^aA^	21.44±1.20^bA^	16.33±0.77^dA^
TAC (mM/dL)	1	16.00±4.00^A^	13.00±3.00^A^	18.00±4.00^A^	19.00±4.00
	2	9.250±1.05^bB^	7.000±1.00^cB^	10.01±1.54^bB^	14.22±0.91^a^

Values with different superscripts (a, b, c&d) within the same raw differ significantly (p < 0.05) among periods; values with superscripts (A&B) differ significantly (p < 0.05) between the two groups

BUN values showed the highest significant (p<,0.05) increase on day 0 than on day +21 and day +42 when compared with day −14 in both groups. In contrast, a significant increase was recorded on day 0 in the control group relative to the hypocalcemia group. Serum Cr concentration revealed a significant increase (p<,0.05) on day −14, then decrease gradually from day 0 till day +42 in both groups, with a significant increase (p<,0.05) on day +42 in the hypocalcemia group. A significant decrease (p<,0.05) in serum Ca level was recorded on day 0, day −14, and day +21, then begins to restore on day +42 in both groups. Furthermore, serum Ca level recorded a significant decrease (p<,0.05) in the hypocalcemia group on day 0 and day −14 in comparison to the control group. Serum P level recorded significant (p<,0.05) decrease being the minimum on day 0 then day −14 and day +21 and begin to restore on day +42 in both groups, with a significant decrease (p<,0.05) in the hypocalcemia group on day 0 and day −14 relative to the control group.

In the control group, there was a significant (p<,0.05) increase in serum vitamin D by day −14, reaching a peak on day 0 before decreasing by day +21 and +42. Meanwhile, non-significant changes were recorded in different periods in the hypocalcemia group, with a significant decrease (p<,0.05) in the hypocalcemia group on day 0 and day −14 in comparison to the control group. Serum cortisol levels revealed a significant (p<,0.05) increase on day 0 followed by day −14, and its level decreased on day +21 and +42 in both the control and hypocalcemia groups, but with the highest value on day 0 in the hypocalcemia group.

**Figure-2:**
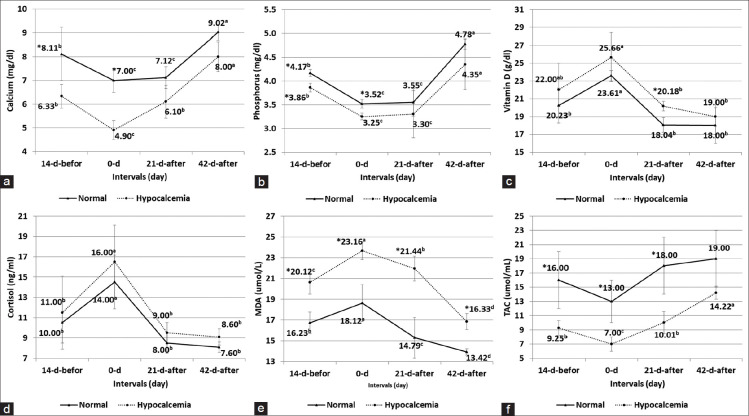
(a) Serum calcium; (b) phosphorus; (c) Vitamin D; (d) cortisol; (e) malondialdehyde (MDA); and (f) total antioxidant capacity (TAC) in the control and hypocalcemia groups (means±SD) on days −14, 0, +21, and +42. Values with different superscripts (a, b, c, and d) differ significantly among periods (p<0.05); values with superscripts (*) differ significantly between the two groups (p<0.05).

Serum MDA levels showed a significant (p<,0.05) increase on day −14, day 0, day +21, and day +42 in the hypocalcemia group when compared to the control group. In the hypocalcemia group, the greatest increase was recorded on day 0, and its values begin to decrease on day +21 and +42 in both groups. In contrast, serum TAC revealed a significant (p<,0.05) decrease greatly on day 0 compared with day −14, then increase again on day +21 and day +42 in the hypocalcemia group. A significant (p<,0.05) increase in serum TAC in different periods was recorded in the control group when compared to the hypocalcemia group, as shown in Figure-2.

## Discussion

Pregnancy and lactation are metabolically stressful phases; therefore, managing the transition period’s metabolic changes is critical [21]. In the present investigation, hypocalcemia was the highest on the elder does (over 3 years) and those with higher parity, due to the inability to absorb and mobilize the stored calcium, making these animals are more susceptible to this ailment [22]. Approximately 65% of the cases occurred in does having more than 2 feti because of the large fetoplacental requirement associated with multiple births [9].

Overall, the observed findings may be explained by the fact that Ca is essential for the contraction of all smooth, cardiac, and skeletal muscles. Low blood calcium concentration reduces food intake, rumen contraction, uterine, and intestinal motility and increases susceptibility to many metabolic and infectious diseases [6]. Our clinical signs were similar to those obtained by Fakour and Hajizadeh [23]. The slight decrease in rectal body temperature was in accordance with Larsen *et al*. [24] who suggested that rectal temperature was directly correlated with the decline in blood calcium as the contraction of the muscles generates body temperature. Tachycardia with a weak pulse observed in the hypocalcemia group also occurred in parallel to the intensity of the hypocalcemia, which may induce a decrease in cardiac contractility, leading to a lower cardiac output [25].

Regarding the effect of hypocalcemia on uterine involution, from a physiological perspective, the uterus is exposed to changes during pregnancy, and glandular tissue develops intensively to accommodate and nourish the developing fetus to term. To rebreed, gross anatomical changes with extensive remodeling in the uterine tissue occur during the puerperium period [26].

The involution of the uterus is significantly delayed by peri-parturient diseases. A retained placenta hinders uterine contraction immediately after the birth, and swollen portions of the uterus result in folding and regional differences in reduction, inhibiting the otherwise rapid involution of the organ. In the present study, the diameter of the uterus decreased sharply within the first 2 weeks in the control group, and uterine involution was completed by day 35 postpartum. These findings are similar to those of Zdunczyk *et al*. [27]. Fernandes *et al*. [28] showed that uterine regression to the standard size was ended within 21 days postpartum in sheep.

In hypocalcemic animals, calcium transfer into the sarcoplasmic reticulum is decreased, causing a reduction of all muscle contraction, inducing dystocia and a retained placenta. Furthermore, the dysregulated calcium metabolism leads to weakness of the genital organs, especially the uterus. This is in agreement with the result of Mohammed and Taher [29] who showed that calcium levels were significantly (p≤0.05) reduced in dystocial animals when compared to normal animals. These findings are consistent with Majeed and Taha [30] who demonstrated that hypocalcemia is considered the leading cause of dystocia in small ruminants. Sedó *et al*. [31] found that the cows that nourished on pasture with concentrations of serum Ca <2 mmoL/L on the birthday had lower first service conception rates (14%) than normocalcemic cows (38%). These results support the present findings where uterine involution was delayed until 42 days postpartum and take a longer time to develop in the hypocalcemia group when compared to controls.

Regarding the hematological profile, the decrease of RBCs, PCV, and Hb concentration on day −14 and day 0 in both groups may be due to the hemodilution and increased plasma volume or due to mobilization of water through the vascular system to the mammary gland as previously reported by El Zein *et al*. [32]. The significant decrease of PCV% in the hypocalcemia group on day −14, day 0, and even on day +21 may be due to the release of antidiuretic hormone as a result of stress. The Hb concentration showed a significant decrease on day 0 and increased significantly up to the 45^th^ day of lactation in the study of Manat *et al*. [33]. The increase of Hb concentration after parturition is attributed to the increased demand for oxygen and greater metabolic rate [34].

The significant (p<,0.05) increase in total WBCs on day −14 reaching the peak on day 0 in both groups may be due to enhanced bone marrow activity and/or pregnancy stress. The non-significant change recorded in WBCs between normal and hypocalcemic goats is consistent with the findings of Martinez *et al*. [5]. The increase in neutrophils on day −14 and day 0 recorded in both groups could be attributed to stress and glucocorticoids secretion, enhancing the mobilization of neutrophils from the body pool into the peripheral circulation [35]. In comparison, the decrease of lymphocytes on day −14 and day 0 in both groups may be due to impaired immune function or stress [33].

The decrease of monocyte and eosinophil on day 0 in both groups is due to cortisol secretion. Fakour and Hajizadeh [23] observed the decrease of white blood cells, lymphocytes, and eosinophils after hypocalcemia induction. Moreover, T-lymphocytes produce eosinophilia, which stimulates eosinophil production in the bone marrow, and eosinophil chemotactic anaphylactic factor, which is chemotactic for eosinophil. This effect is mediated during stressful situations by the anterior pituitary and adrenocortical hormones [36].

The observed increase in serum glucose concentration on day 0 in both groups could be attributed to the increased cortisol levels. Ingvartsen and Andersen [37] revealed that the gradual increase of cortisol 5 days before delivery stimulates hepatic gluconeogenesis and increases circulating glucose. In contrast, hypoglycemia at parturition and the 1^st^ week of lactation in high-producing dairy goats was reported by Wankhade *et al*. [21]. This is due to the utilization by the growing feti and high demand for milk lactose synthesis.

The decrease of serum total protein and globulin concentration recorded on day −14 and day 0 was consistent with Soares *et al*. [38], and it could be due to the usage of maternal amino acids for feti proteins synthesis and fetal growth [39] or for the preparation for milk synthesis, which begins about 3-4 weeks before parturition [40]. Drainage and migration of plasma globulins, particularly g-globulin for colostrum synthesis postpartum, are the primary cause of globulins reduction [41]. Our findings are in agreement with Balikci *et al*. [42] who recorded no observed changes in serum albumin during different stages in both groups. More specifically, a non-significant change was detected in serum AST and ALT enzymes, indicating that the liver was not clinically affected. Higher serum ALP activity on day −14 and day 0 of kidding when compared with postpartum, may be attributed to stressful condition as ALP is a physical stress indicator [43], or due to the increased placental production of this enzyme [44].

The decrease of BUN on day −14 and increased levels on days +21 and +42 is consistent with El Zein *et al*. [32] and may be due to increased protein catabolism in the body by the elevated concentration of cortisol. Significantly higher Cr concentration on day −14 compared to postpartum might be due to a high level of cortisol [33]. The changes in Cr concentration are linked to the mobilization of maternal protein for fetal musculature development and the removal of the fetal organic residues.

A significant decrease in serum Ca levels was recorded on day 0 and day −14, and day +21 before they began to restore by day +42 in both groups. These changes may be due to the increased Ca demand mineralization of fetal skeleton and colostrum and milk production. Kimura *et al*. [45] proposed that the great demand for calcium prepartum contributes to decreased intracellular Ca^2+^ stores even before hypocalcemia progresses. In the present study, serum phosphorus levels showed a significant decrease on day 0 until day −14, when they begin to restore by day +42 in both groups. In contrast, Soares *et al*. [38] observed no P level change during the transition period. The change in the plasma P concentration is related to parathormone changes. The active form of vitamin D 1,25 (OH)2D3 is essential to stimulate the intestinal absorption and renal reabsorption of Ca [11] and maintain its average level in blood. A significant decrease of vitamin D during pregnancy in hypocalcemic goats is consistent with Goff *et al*. [46]. They suggested that the cause of milk fever is due to insufficient or delayed 1,25-(OH)_2_D production. Reactive oxygen metabolites inactivate cytochrome P450 enzymes [47], which are necessary for the hydroxylation of cholecalciferol in the 1 and 25 positions [48], subsequently inhibits 1,25-(OH)_2_D production. The current research revealed a significant increase in vitamin D during the post-parturient period in the control group. Toverud *et al*. [49] exhibited an elevation of 1, 25(OH)_2_ D3 2- to 3-fold during late gestation in most species.

The significantly increased cortisol levels on days −14 and 0 may be due to increased secretion of fetal adrenocorticotropic hormone in the late stage of pregnancy. In addition, the increased cortisol enters the maternal circulation and induces parturition by activating prostaglandin F2a production [50].

From a physiological perspective, reactive oxygen species (ROS) are counteracted by the antioxidant system [51]. The imbalance between increased ROS production and reduced antioxidant system capacity induces oxidative stress [52].

MDA is produced during the peroxidation of polyunsaturated fatty acids by the action of ROS because of the depletion of antioxidant systems [53]. High production animals are more likely to be exposed to free radical peroxidation processes and MDA production [54]. The significant increase of MDA levels on days −14 and 0, which was greater and obviously in the hypocalcemia group, agrees with the results of Colakoglu *et al*. [55] who reported a gradual increase in MDA levels from −21 to calving days then decreased after calving. Calving and lactation are considered a cause of oxidative stress and affect the blood MDA levels. Metabolic requirements increase during the prepartum period for fetal development and colostrogenesis, and, therefore, require more energy and oxygen and suffer increased ROS production [55]. The continuous increase of MDA postpartum in current hypocalcemic goats could be due to greater energy and oxygen requirements than prepartum.

Total antioxidant activity (TAC) is considered a cumulative action of all antioxidants present in serum and body fluids. It describes the dynamic equilibrium between pro-oxidants and antioxidants in the plasma compartment [56]. Indeed, TAC is a helpful tool to measure stress in ruminants [57]. Higher antioxidants concentrations contributed to delaying or reducing the protein oxidation processes [58]. The current decrease of antioxidant activity on days −14 and 0 is consistent with Kumar *et al*. [59]. They attributed this decrease to increased energy demand and lipolysis of body reserves, which leads to elevated free radical production and reduced antioxidant activity.

## Conclusion

Does were susceptible to hypocalcemia during peri-parturient periods. Peri-parturient hypocalcemia causes metabolic and oxidative stress in does as well as the retardation of uterine involution in postpartum periods. Biochemical analysis during peri-parturient is helpful for the diagnosis of metabolic disorders in does.

## Authors’ Contributions

All authors contributed to the study design and drafted the manuscript. NEA and AAA collected the samples and animals follow-up. YHB and AB analyzed data and reviewed results. All authors read and approved the final manuscript.
